# Aging Reduces Insulin Clearance in Mice

**DOI:** 10.3389/fendo.2021.679492

**Published:** 2021-05-12

**Authors:** Carine Marmentini, Gabriela M. Soares, Gabriela A. Bronczek, Silvano Piovan, Cecília E. Mareze-Costa, Everardo M. Carneiro, Antonio C. Boschero, Mirian A. Kurauti

**Affiliations:** ^1^ Laboratory of Endocrine Pancreas and Metabolism, Obesity and Comorbidities Research Center (OCRC), University of Campinas (UNICAMP), Campinas, Brazil; ^2^ Department of Physiological Sciences, Biological Sciences Center, State University of Maringa (UEM), Maringa, Brazil

**Keywords:** CEACAM1, hepatic insulin clearance, hyperinsulinemia, insulin-degrading enzyme, insulin secretion, insulin sensitivity

## Abstract

Hyperinsulinemia is frequently associated with aging and may cause insulin resistance in elderly. Since insulin secretion and clearance decline with age, hyperinsulinemia seems to be maintained, primarily, due to a decrease in the insulin clearance. To investigate these aging effects, 3- and 18-month-old male C57BL/6 mice were subjected to intraperitoneal glucose and insulin tolerance tests (ipGTT and ipITT) and, during the ipGTT, plasma c-peptide and insulin were measure to evaluate *in vivo* insulin clearance. Glucose-stimulated insulin secretion in isolated pancreatic islets was also assessed, and liver samples were collected for molecular analyses (western blot). Although insulin sensitivity was not altered in the old mice, glucose tolerance, paradoxically, seems to be increased, accompanied by higher plasma insulin, during ipGTT. While insulin secretion did not increase, insulin clearance was reduced in the old mice, as suggested by the lower c-peptide:insulin ratio, observed during ipGTT. Carcinoembryonic antigen-related cell adhesion molecule-1 (CEACAM1) and insulin-degrading enzyme (IDE), as well as the activity of this enzyme, were reduced in the liver of old mice, justifying the decreased insulin clearance observed in these mice. Therefore, loss of hepatic CEACAM1 and IDE function may be directly related to the decline in insulin clearance during aging.

## Introduction

Aging is commonly associated with insulin resistance and hyperinsulinemia (1, 2). Although it is hypothesized that insulin resistance may cause a compensatory hyperinsulinemia (3), it has been demonstrated that hyperinsulinemia downregulates insulin receptors at the cellular membrane and disrupts post-receptor intracellular signaling in its target cells, inducing insulin resistance (4, 5). Thus, it remains unclear whether insulin resistance or hyperinsulinemia comes first during the aging process.

In mice, genetic ablation of insulin gene (*Ins2* ^+/-^) reduced the circulating levels of this hormone, and this reduction preserved their insulin sensitivity as they aged, compared with their controls (6). It suggests that hyperinsulinemia might induce insulin resistance during aging. Therefore, to investigate the mechanisms whereby circulating insulin levels increase with age it is important to find new strategies to counteract this age-related disorder.

Plasma insulin levels are determined by insulin secretion, and its removal from the circulation, known as insulin clearance. Thus, increased insulin secretion and/or decreased insulin clearance could contribute to hyperinsulinemia during aging. While several studies have reported decreased insulin secretion in aged rodents and humans (7, 8), others have reported decreased insulin clearance in elderly (9, 10). These latter data suggest that age-related hyperinsulinemia could be explained, primarily, by a reduction in the insulin clearance. Therefore, to better understand the effects of aging upon insulin clearance, the molecular mechanisms involved in this reduction should be investigated.

Insulin clearance has, basically, two components: hepatic and extrahepatic clearance. Since the hepatic insulin clearance can remove about 50 to 80% of insulin secreted, during its first passage through the liver (11), we focus on this component. In the liver, this process is initiated when insulin binds to its receptor (IR). After IR is activated by insulin, an important protein that promotes receptor-mediated insulin internalization, namely carcinoembryonic antigen-related cell adhesion molecule 1 (CEACAM1), is activated and it associates with insulin-IR complex, targeting this complex to clathrin-coated pits/vesicles, triggering the endocytosis process. In the early endosome, insulin-IR complex is destabilized and the IR may be recycled to the cellular membrane, *via* retro-endocytosis, while insulin is cleaved by the major enzyme responsible for its degradation, the insulin-degrading enzyme (IDE) (11–13). Although IDE have been considered an important enzyme involved with insulin clearance, recent studies have demonstrated that liver-specific ablation of IDE (L-IDE-KO) did not affect insulin clearance in mice (14, 15), suggesting that other molecular mechanisms may play an important role in this process. Indeed, mice with global null mutation or with liver-specific inactivation of Ceacam1 gene display hyperinsulinemia due to their impaired insulin clearance, which in turn induces insulin resistance in these mice (16, 17).

Here, we evaluated the glucose homeostasis, insulin secretion and hepatic insulin clearance in 3- and 18-month-old mice. We also investigated whether the effects of aging upon hepatic insulin clearance were related to changes in the CEACAM1 and IDE expression, as well as IDE activity, in the liver of these mice.

## Material & Methods

### Animals

Twenty male C57BL/6 mice from the University of Campinas (UNICAMP) facilities were housed collectively (5 animals per cage) and maintained under a light-dark cycle (12 h light and 12 h dark) with a controlled humidity and temperature until 3- (control group, CTL, n=10) or 18-months-old (old group, OLD, n=10). These mice were allowed to freely drink tap water and feed a standard chow diet. The described experimental procedures were approved by the Committee on Ethics in the Use of Animals of the UNICAMP (CEUA-UNICAMP, approval number 4659‐1/2017), and were conduct in accordance with the last revision of the National Institutes of Health (NIH) guide for the care and use of laboratory animals.

### Intraperitoneal Glucose and Insulin Tolerance Tests (ipGTT and ipITT)

To test glucose tolerance, mice were restricted to food during 10 h before they receive an intraperitoneal administration of 1 g × kg^-1^ glucose load. Their blood glucose was measured before (0 min) and 15, 30, 60 and 120 min after glucose load administration, from the tip of their tails using a blood glucose meter (Accu-chek^®^, Roche, Basileia, Switzerland). To test insulin tolerance, mice were restricted to food during 2 h before they receive an intraperitoneal administration of 0.75 U × kg^-1^ insulin (Humulin R; Eli Lilly, Indianapolis, IN, USA), and their blood glucose was measured before (0 min) and 5, 10, 15, 20, 25, 30 and 60 min after insulin administration.

### *In Vivo* Insulin Clearance

The insulin clearance of mice was evaluated calculating plasma c-peptide:insulin ratio, during the ipGTT, as previously described (18). To this purpose, blood samples were collected from the tip of the tail before (0 min) and after 15 and 60 min glucose load administration. The blood samples were centrifuged (1100 g, during 15 min at 4°C) to obtain plasma, which were stored at -80°C to posterior c-peptide and insulin measurements. These hormones were measured using specifics enzyme‐linked immunosorbent assay (ELISA) kits according to the manufacturer’s instructions (Mouse C-Peptide ELISA Kit Catalog # 90050 and Ultra-Sensitive Mouse Insulin ELISA Kit Catalog # 90080, Crystal Chem, Elk Grove Village, IL, USA).

### Glucose-Stimulated Insulin Secretion in Isolated Pancreatic Islets

All mice were anesthetized with isoflurane and killed by decapitation to dissect and collect tissues, such as the pancreas, which were digested with collagenase to isolate pancreatic islets, as described before (19). Five islets from each mouse were used to assess the glucose-stimulated insulin secretion as previously described (20) with minor modifications. After 1 h preincubation in Krebs-Ringer bicarbonate (KRB) buffer containing 0.3% bovine serum albumin (BSA) and 5.6 mmol × l^-1^ glucose (95% O2, 5% CO2, pH 7.4, at 37°C), the islets were incubated for an additional hour in the same buffer containing 0.3% BSA and 2.8 or 11.1 mmol × l^-1^ glucose. After this incubation, the supernatants were collected to access insulin secretion and the remaining islets were homogenized in an alcohol-acid solution to measure total insulin content using the Ultra-Sensitive Mouse Insulin ELISA Kit (Catalog # 90080, Crystal Chem, Elk Grove Village, IL, USA).

### Western Blot Analyses

Liver samples were also collected to evaluate protein expression by western blot as previously described (21). In this study, the primary antibodies and their respective dilutions used, in this study, were as follow: anti‐IDE 1:500 (Catalog ab32216, Abcam, Cambridge, UK); anti-CEACAM1 1:500 (Catalog 14771, Cell Signaling, Danvers, MA, USA); and anti-α-Tubulin 1:30000 (Catalog T5168, Sigma-Aldrich, St Louis, MO, USA).

### IDE Activity Measurements

Liver IDE activity was measured using the SensoLyte 520 IDE Activity Assay Kit according to the manufacturer’s instructions (Catalog AS‐72231; AnaSpec, Fremont, Canada). Total IDE activity was calculated as described before (18) and normalized per μg of total protein content determined using the Bio-Rad Protein Assay Dye Reagent Concentrate (Catalog #5000006, Bio-Rad, Hercules, CA, USA).

### Statistics

Normal distribution of the data and homogeneity of variance were tested, and to compare data from CTL and OLD groups (CTL vs OLD) Student’s unpaired t-test was applied. These statistical analyses were performed using Prism software version 8.0.1 for Windows (GraphPad Software, La Jolla, CA, USA). The sample size (n) used for the statistical analysis of each group was described in the figure’s legends. All data were presented as the mean ± standard deviation (SD) and were considered significantly different if the p-value was equal or lower than 0.05 (p ≤ 0.05).

## Results

### Aging Did Not Change Fasting Blood Glucose and Plasma Insulin Levels

Eighteen-month-old (OLD) mice had increased body weight and reduced gastrocnemius muscle pad without change in the perigonadal fat pad, compared with 3-month-old (CTL) mice, as shown in the **Table 1**. In addition, fasting blood glucose and plasma insulin levels were not different between the groups.

**Table 1:** Metabolic parameters of control and old mice.

Metabolic parameters (units)	CTL	OLD
Body weight (g)	23.81 ± 0.642 (n=10)	29.47 ± 1.173 (n=10)*
Skeletal muscle pad (% of body weight)	0.578 ± 0.041 (n=10)	0.470 ± 0.023 (n=10)***
Fat pad (% of body weight)	1.058 ± 0.160 (n=10)	1.121 ± 0.352 (n=10)
Fasting glycemia (mg × dl^-1^)	107.1 ± 10.52 (n=10)	99.7 ± 15.85 (n=10)
Fasting insulinemia (ng × ml^-1^)	0.172 ± 0.029 (n=10)	0.175 ± 0.057 (n=10)

*p ≤ 0.05 and ***p ≤ 0.001 vs CTL (Student’s unpaired t-test).

### Aging Increased Glucose Tolerance Without Changing Insulin Sensitivity

**Figure 1:**
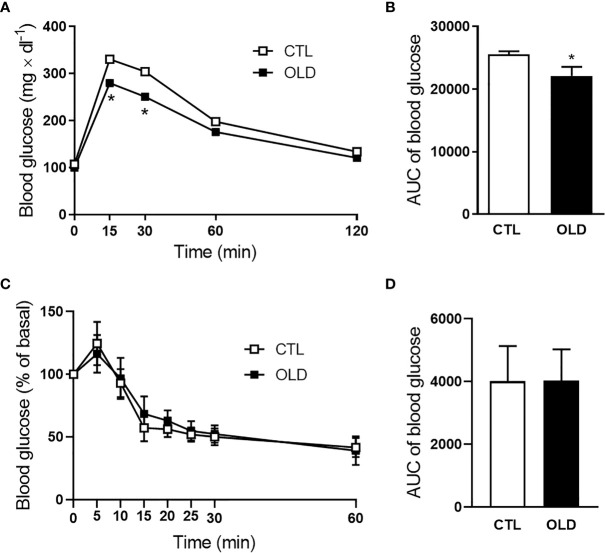
Effect of aging upon glucose and insulin tolerance. **(A)** Blood glucose and **(B)** its AUC before (0 min) and 15, 30, 60 and 120 min after 1g × kg^-1^ intraperitoneal glucose administration (ipGTT, n = 10 CTL and 10 OLD). **(C)** Blood glucose and **(D)** its AUC before (0 min) and 5, 10, 15, 20, 25, 30 and 60 min after 0.75U × kg^-1^ intraperitoneal insulin administration (ipITT, n = 9 CTL and 9 OLD). CTL, 3-month-old mice; and OLD, 18-month-old mice. Data are presented as the mean ± standard deviation (SD). Student’s unpaired t-test was used to compare the groups (*p ≤ 0.05 *vs* CTL).

To evaluate glucose homeostasis, intraperitoneal glucose and insulin tolerance tests (ipGTT and ipITT) were performed. During the ipGTT, OLD mice presented decreased blood glucose levels at 15 and 30 min (**Figure 1A**). Also, the area under the curve (AUC) was lower, compared with CTL mice (**Figure 1B**). Although the OLD mice displayed increased glucose tolerance, their insulin sensitivity was similar to that observed in the controls (**Figures 1C, D**).

### Aging Decreased Hepatic Insulin Clearance

**Figure 2:**
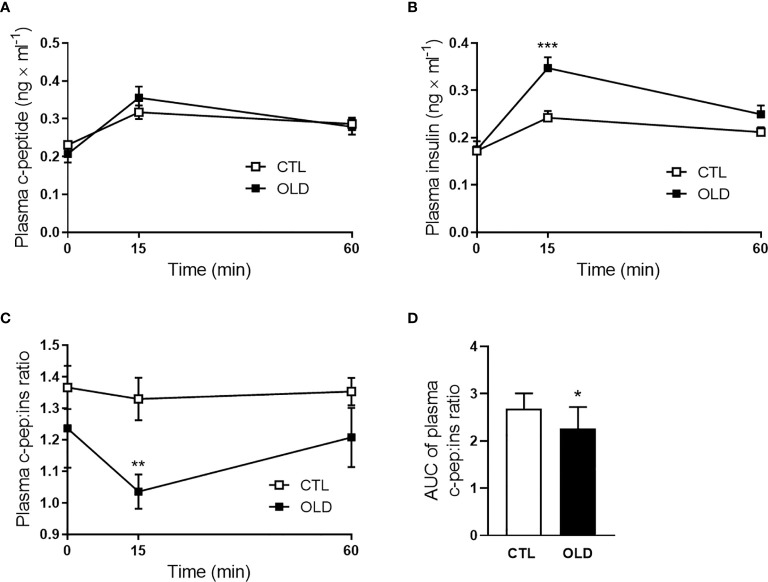
Effect of aging upon plasma c-peptide:insulin ratio (insulin clearance) during the ipGTT. **(A)** Plasma c-peptide, **(B)** insulin, and **(C)** c-peptide:insulin ratio at 0, 30 and 60 min after 1g × kg^-1^ glucose load administration, and **(D)** AUC of plasma c-peptide:insulin ratio (n = 10 CTL and 9 OLD). CTL, 3-month-old mice; and OLD, 18-month-old mice. Data are presented as the mean ± standard deviation (SD). Student’s unpaired t-test was used to compare the groups (*p ≤ 0.05, **p ≤ 0.00 and ***p ≤ 0.001 *vs* CTL).

During the ipGTT, blood samples were collected and the plasma was used to measure c-peptide and insulin levels at 0, 15 and 30 min after the glucose load (**Figures 2A, B**). Although plasma c-peptide levels were similar between groups, plasma insulin levels were significantly higher in the OLD at 15 min, compared with CTL group, provoking a reduction in the c-peptice:insulin ratio at this time point (**Figure 2C**). It seems that insulin secretion was not altered, since plasma c-peptide was similar between groups, but the hepatic insulin clearance was reduced in the OLD group, as judged by their lower AUC of plasma c-peptide:insulin ratio, compared with the CTL’s ratio (**Figure 2D**).

### Aging Did Not Alter Glucose-Stimulated Insulin Secretion in Isolated Pancreatic Islets

**Figure 3:**
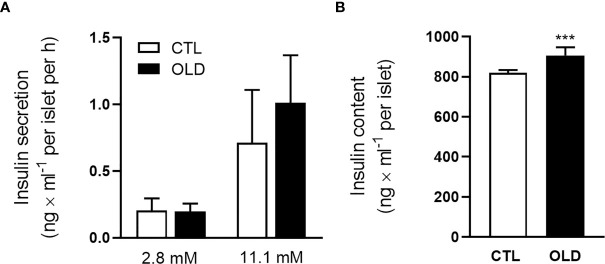
Effect of aging upon glucose-stimulated insulin secretion in isolated pancreatic islets. **(A)** Insulin secretion per islet after 1 h incubation with 2.8 or 11.1 mmol × l^-1^ glucose. **(B)** Total insulin content per islet (n = 10 CTL and 10 OLD). CTL, 3-month-old mice; and OLD, 18-month-old mice. Data are presented as the mean ± standard deviation (SD). Student’s unpaired t-test was used to compare the groups (***p ≤ 0.001 *vs* CTL).

Corroborating the similar plasma c-peptide levels between the groups, during the ipGTT, glucose-stimulated insulin secretion was not significantly different in isolated pancreatic islets (**Figure 3A**), although insulin content was higher in the OLD, compared with CTL group (**Figure 3B**).

### Aging Decreased Hepatic CEACAM1 and IDE Expression

**Figure 4:**
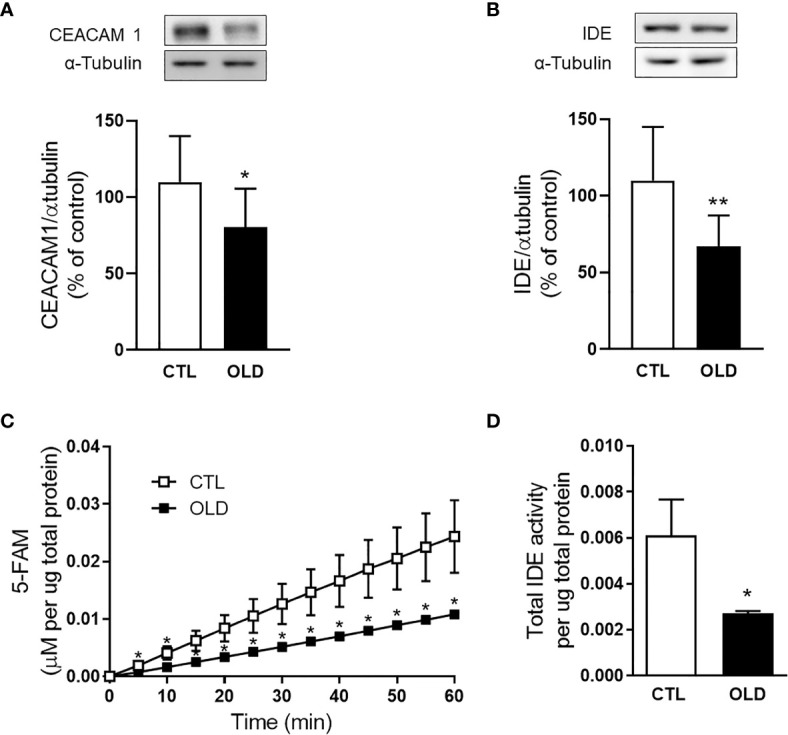
Effect of aging upon hepatic CECAM1 and IDE expression, and upon hepatic IDE activity. Protein expression of **(A)** CEACAM1 and **(B)** IDE in the liver from the mice and its representative immunoblotting images (CEACAM1, n = 10 CTL and 10 OLD; IDE, n = 10 CTL and 10 OLD). **(C)** Kinetics of the IDE activity assay in liver of mice. Fluorescent intensity at Ex/Em = 490/520 nm was continuously recorded, every 5 min, during 60 min. 5‐FAM concentration was calculated using a standard curve and normalized per μg of total protein. **(D)** IDE activity was calculated as previously described (18) and normalized per μg of total protein in the liver (n = 10 CTL and 10 OLD). CTL, 3-month-old mice; and OLD, 18-month-old mice. Data are presented as the mean ± standard deviation (SD). Student’s unpaired t-test was used to compare the groups (*p ≤ 0.05 and **p ≤ 0.001 *vs* CTL).

To investigate the molecular mechanism whereby aging decreases hepatic insulin clearance, we evaluate the expression of proteins involved with this process. The expression of the transmembrane protein involved with the endocytosis of the insulin-IR complex, CEACAM1, was decreased in the liver from the OLD mice compared with controls (**Figure 4A**). Also, IDE, an important enzyme that degrades insulin, had its expression (**Figure 4B**) and activity (**Figures 4C, D**) reduced in the liver from the OLD, compared with CTL mice.

## Discussion

Hyperinsulinemia is related to aging and may be the consequence of an increase in insulin secretion and/or a decrease in its clearance. In our previous study, while insulin secretion was increased, insulin clearance did not change in 10-month-old mice compared with 3-month-old mice (22). Here, 18-month-old mice displayed similar insulin secretion, whereas hepatic insulin clearance was lower to that found in the 3-month-old mice. These data suggest that with advancing age, β-cells from pancreatic islets may lose their ability to maintain a higher insulin secretion. To compensate, hepatic insulin clearance is reduced, probably due to a lower expression of CEACAM1 and IDE, associated with a decreased IDE activity, in the liver.

Although several studies have demonstrated impairment on glucose tolerance with age (23, 24), here, the OLD mice had improved glucose tolerance (which might be explained by the elevated plasma insulin level, as shown in **Figure 2B**), and had no change in the insulin sensitivity (**Figures 1C, D**). These data contrast to those reported in our previous study using 10- and 3-month-old mice (22). Ten-month-old mice displayed glucose intolerance, insulin resistance and hyperinsulinemia, compared with their controls. Thus, it seems that 18-month-old mice are metabolic different from 10-mont-old mice. Indeed, the body weight of 10-mont-old mice is higher than 18-month-old mice (36.05 ± 1.546 g *vs* 29.47 ± 1.173 g). Also, the perigonadal fat pad weight (% of body weight) seemed to be increased in the 10-month-old mice compared with their controls in the previous study (CTL = 1.738 ± 0.238 g *vs* OLD = 2.861 ± 0.495 g, p = 0.075), whereas here, this increase was not observed (CTL = 1.058 ± 0.160 g *vs* OLD =1.121 ± 0.352 g, p = 0.610). These differences may explain the glucose intolerance and insulin resistance observed in the 10-month-old mice used in our previous study, compared with the 18-month-old mice used here, since the increase in visceral fat pad may raise the risk for insulin resistance (25, 26).

The paradoxical normal insulin sensitivity, found in the OLD mice, led us to ask whether age-insulin resistance is an obligatory finding. We found the answer in studies with centenarians (90-100 years old) that have a preserved insulin action compared with aged subjects (<80 years old) (27). These studies show that age-related insulin resistance is not an obligatory finding in the elderly, and this may be found in other species, including rodents, as we described here.

Although age-related hyperinsulinemia was previously associated with increased insulin secretion (22, 28), here, insulin secretion in the OLD mice was similar to that found in their controls. It is possible that, in these 18-month-old mice, β-cells are in decline of their function, and the compensatory hypersecretion of insulin, that probably have occurred earlier, may not be observed at this stage. Decreased expression of the glucose transporter 2 (GLUT2) (29), decreased Ca^2+^ influx (18), mitochondrial dysfunction (30) and chronic low-grade inflammation (31), observed in aged β-cells, might be the molecular mechanisms involved with the decline in insulin secretion that occurs with age.

Since insulin secretion was not altered in the OLD mice compared with their controls (**Figure 3A**), the hyperinsulinemia observed in the former, after a glucose load (**Figure 2B**), could be due to an impaired hepatic insulin clearance as suggested by the lower c-peptide:insulin ratio, during the ipGTT (**Figures 2C, D**), in the OLD mice, compared with controls.

It is important to be aware that the c-peptide:insulin ratio can be used to measure hepatic insulin clearance when the c-peptide clearance does not change between the experimental groups. As observed in isolated pancreatic islets, insulin secretion in the OLD was not different from that found in the CTL group (**Figure 3A**). Since c-peptide is co-secreted with insulin at 1:1 molar ratio, the secretion of this hormone was not different between the groups. Considering this similar secretion of c-peptide, and the similar c-peptide kinetic, observed during the ipGTT (**Figure 2A**), we can assume that the c-peptide clearance does not change between the groups, validating our hepatic insulin clearance measurements.

During the ipGTT (**Figure 2B**), we observed lower hepatic insulin clearance only 15 min after the glucose load. This data suggests that this impairment only emerges during a glucose stimulation. We believe that in the fasting state, the liver of the OLD mice can properly handle a small amount of insulin secreted by the pancreas. However, when glucose stimulates insulin secretion, the liver of the OLD mice cannot handle the excess of insulin that reaches this organ, as the liver of the CTL mice.

Although several studies have considered IDE as the major enzyme involved with hepatic insulin clearance, recent studies using L-IDE-KO mice suggest that other molecular mechanisms must be more important to modulate hepatic insulin clearance, such as CEACAM1 expression (14–16). Here, 18-month-old mice that displayed lower hepatic insulin clearance, had a decreased CEACAM1 expression in the liver, compared with their controls (**Figure 4A**), similar to the data found in 18-month-old rats (**Supplementary Figure S1**). Corroborating these data, the hepatic expression of CEACAM1 did not decrease when insulin clearance was not significantly changed in the 10-month-old mice (**Supplementary Figure S2**).

During the process of hepatic insulin clearance, CEACAM1 is phosphorylated at specific tyrosine residue (Tyr 488) by the activated insulin receptor. This phosphorylation allows CEACAM1 to associate with insulin-IR complex, *via* Shc (SH2-containing adapter protein), targeting this complex to clathrin-coated pits/vesicles by interaction with the adaptor protein-2 (AP2) complex (13, 32), thereby triggering the endocytosis process. Therefore, although we evaluated CEACAM1 expression, it is important that further studies also investigate its activation by measuring the tyrosine phosphorylation of this protein in the liver of aged rodents.

In addition to changes in CEACAM1, changes in IDE function might be also associated with alterations in hepatic insulin clearance. Previously, in 10-month-old mice, lower hepatic IDE activity was compensated by the higher expression of this enzyme, maintaining insulin clearance similar to that found in the 3-month-old mice (33). However, considering that 12‐month-old rats (older than 10-month-old) (34) showed a decrease in the hepatic IDE expression compared with their young controls, we speculated that this could also occur in the 18-month-old mice. As expected, we confirmed this effect of aging (**Figure 4B**), which might contribute to the decreased hepatic insulin clearance observed in these OLD mice.

Even though, the contribution of IDE for the modulation of insulin clearance remains controversial. It was suggested that this enzyme in the liver contributes to modulate insulin sensitivity (14, 15). Indeed, pathological conditions related with insulin resistance, such as obesity and type 2 diabetes, are frequently associated with lower hepatic IDE expression and activity (18, 35, 36), while physical exercise, which improves insulin sensitivity, is associated with higher hepatic IDE expression and activity (37–39). In line with these data, insulin resistance observed in 10-month-old mice was accompanied by a lower hepatic IDE activity compared with their young controls (22). However, in the present study, the reduction in the IDE activity in the liver from 18-month-old mice (**Figures 4C, D**), was not associated with insulin resistance. It is possible that the impairment on hepatic IDE activity might precede insulin resistance, but to confirm this hypothesis a time-course study is necessary.

Taking into account all data from 10- and 18-month-old mice, one effect of aging is consistent, hepatic IDE activity reduces with age. This effect was also observed in 18-month-old rats (**Supplementary Figure S3**) and this may be involved with an impaired glucose homeostasis, frequently observed in aged subjects. Previously, we suggested that an increased expression of the inducible nitric oxide synthase (iNOS), observed in the liver from 10-month-old mice, should be linked to the reduction in the hepatic IDE activity, because it was reported that nitric oxide (NO) inhibits insulin degradation by IDE (40, 41). Here, the expression of iNOS was not increased, in fact, it was decreased in the liver from 18-month-old mice compared with controls (**Supplementary Figure S4**), suggesting that other molecular mechanisms must be involved in the impairment on IDE function in the liver of these OLD mice (35, 42).

In summary, insulin clearance reduces with age and this may contribute to age-related hyperinsulinemia. Although previous studies suggest that IDE is not involved in the modulation of hepatic insulin clearance, in control and obese mice, our finds suggest that during aging this enzyme might have a role in this modulation, as well as, the CEACAM1. Therefore, to investigate the molecular mechanisms whereby aging reduces IDE and CEACAM1 function, in the liver, might be helpful to understand how insulin clearance is affected by age.

## Data Availability Statement

The raw data supporting the conclusions of this article will be made available by the authors, without undue reservation.

## Ethics Statement

The animal study was reviewed and approved by Committee on Ethics in the Use of Animals of the UNICAMP (CEUA-UNICAMP, approval number 4659‐1/2017), Sao Paulo, Brazil.

## Author Contributions

Conceptualization, MK. Methodology, MK, CM, and GS. Formal Analysis, MK and CM. Investigation, MK, CM, GS, GB, and SP. Resources, CM-C, AB, and EC. Data Curation, CM. Writing – Original Draft Preparation, MK. Writing – Review & Editing, MK and AB. Visualization, MK. Supervision, MK. Project administration, MK. Funding Acquisition, AB, EC, and CM-C. All authors contributed to the article and approved the submitted version.

## Funding

This research was founded by the São Paulo Research Foundation (FAPESP, grant numbers 13/07607-8, 15/12611-0, 17/06475-1 and 18/24368-0). The funder had no role in study design; in the collection, analysis, and interpretation of data; in the writing of the report; and in the decision to submit the manuscript for publication.

## Conflict of Interest

The authors declare that the research was conducted in the absence of any commercial or financial relationships that could be construed as a potential conflict of interest.
