# Supplementary material for: Aging Reduces Insulin Clearance in Mice
Source: Front Endocrinol (Lausanne). 2021 May 12;12:679492. doi: 10.3389/fendo.2021.679492 (PMC8150109; doi:10.3389/fendo.2021.679492)
Supplement: Supplementary file 1 [file DataSheet_1.docx]

Supplementary Material

# Supplementary Figures


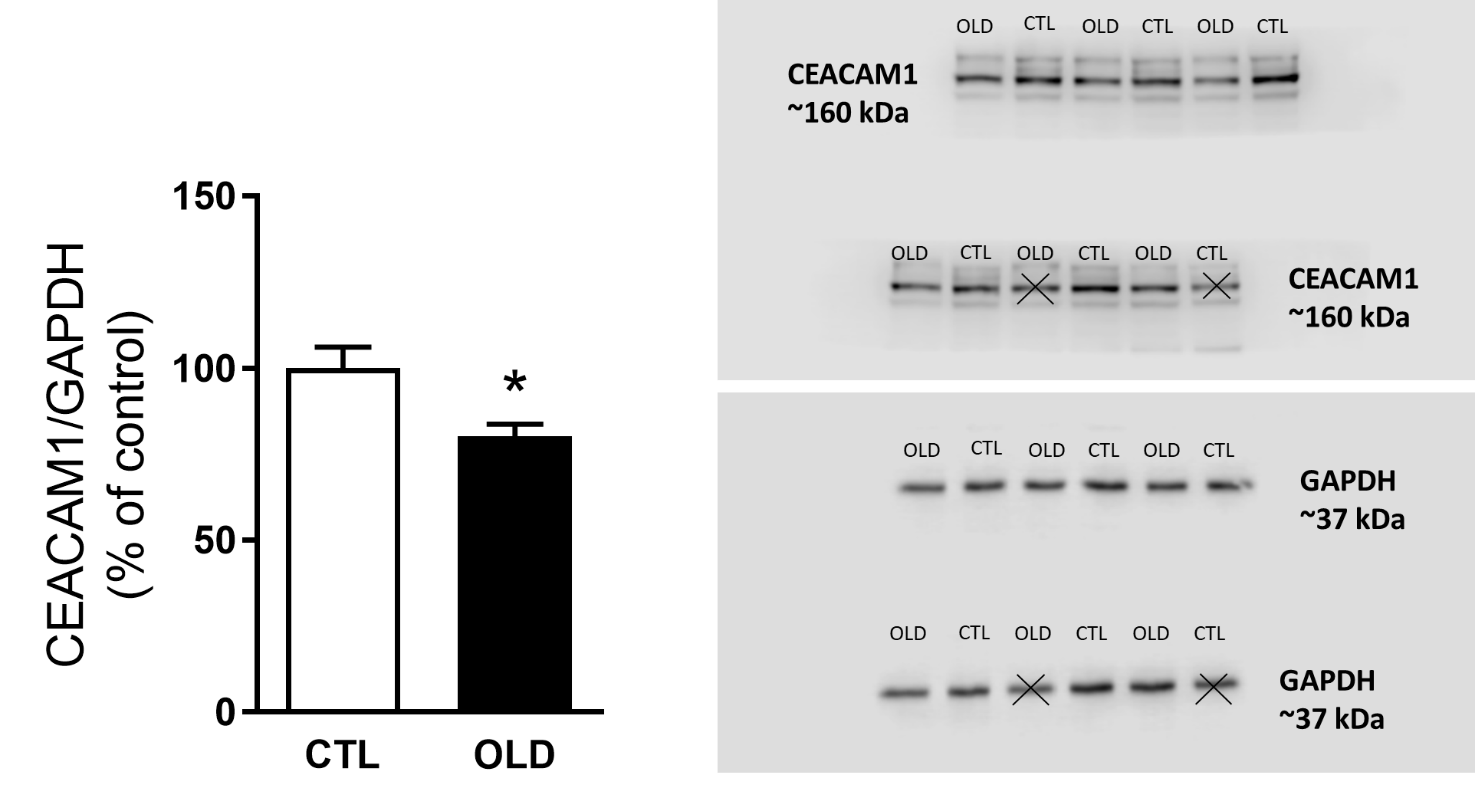


**Supplementary Figure 1.** CEACAM1 expression in the liver from 3- and 18-month-old rats. Protein expression of carcinoembryonic antigen-related cell adhesion molecule-1 (CEACAM1) in the liver and its full unedited immunoblotting images (n = 5 CTL and 5 OLD). CTL, 3-month-old rats; and OLD, 18-month-old rats. Data are presented as the mean ± standard deviation (SD). Student’s unpaired t-test was used to compare the groups (*p ≤ 0.05 *vs* CTL).

**
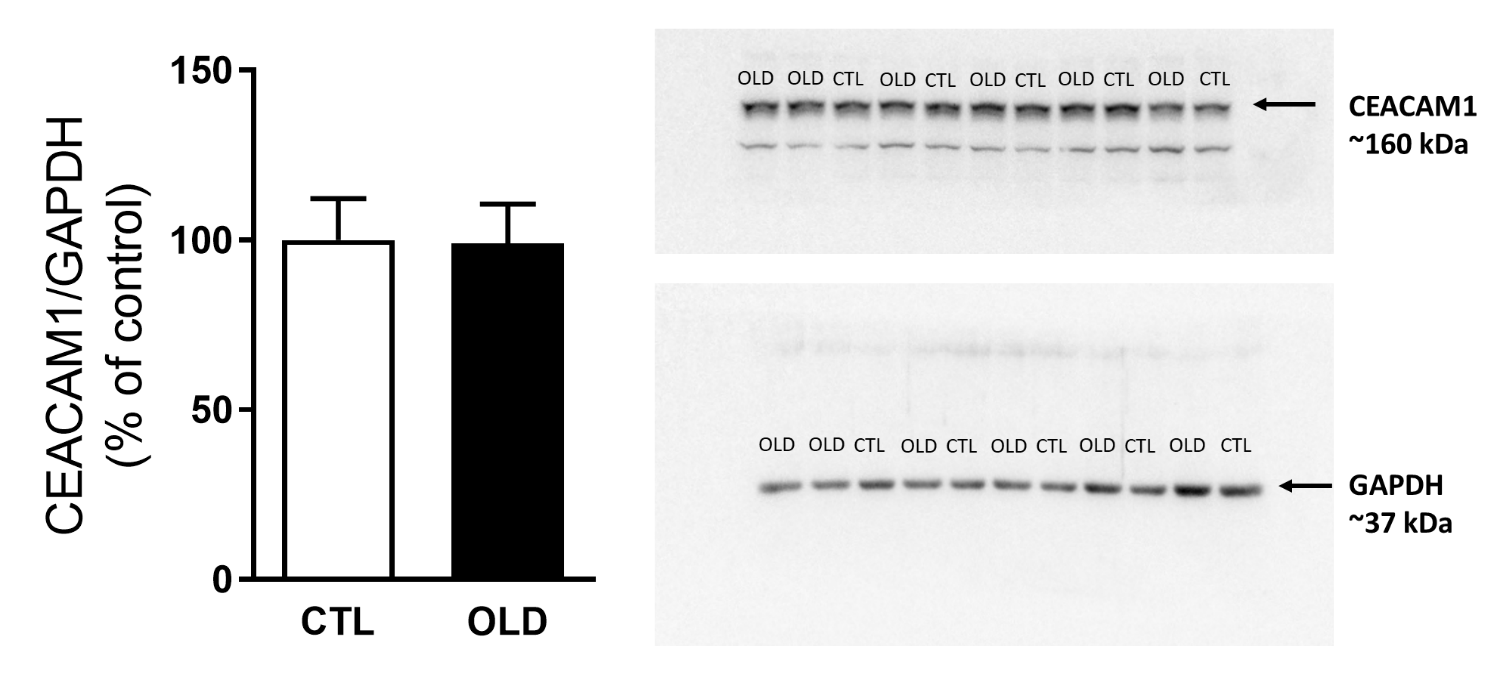
**

**Supplementary Figure 2.** CEACAM1 expression in the liver from 3- and 10-month-old mice. Protein expression of carcinoembryonic antigen-related cell adhesion molecule-1 (CEACAM1) in the liver and its full unedited immunoblotting images (n = 5 CTL and 6 OLD). CTL, 3-month-old mice; and OLD, 10-month-old mice. Data are presented as the mean ± standard deviation (SD). Student’s unpaired t-test was used to compare the groups.


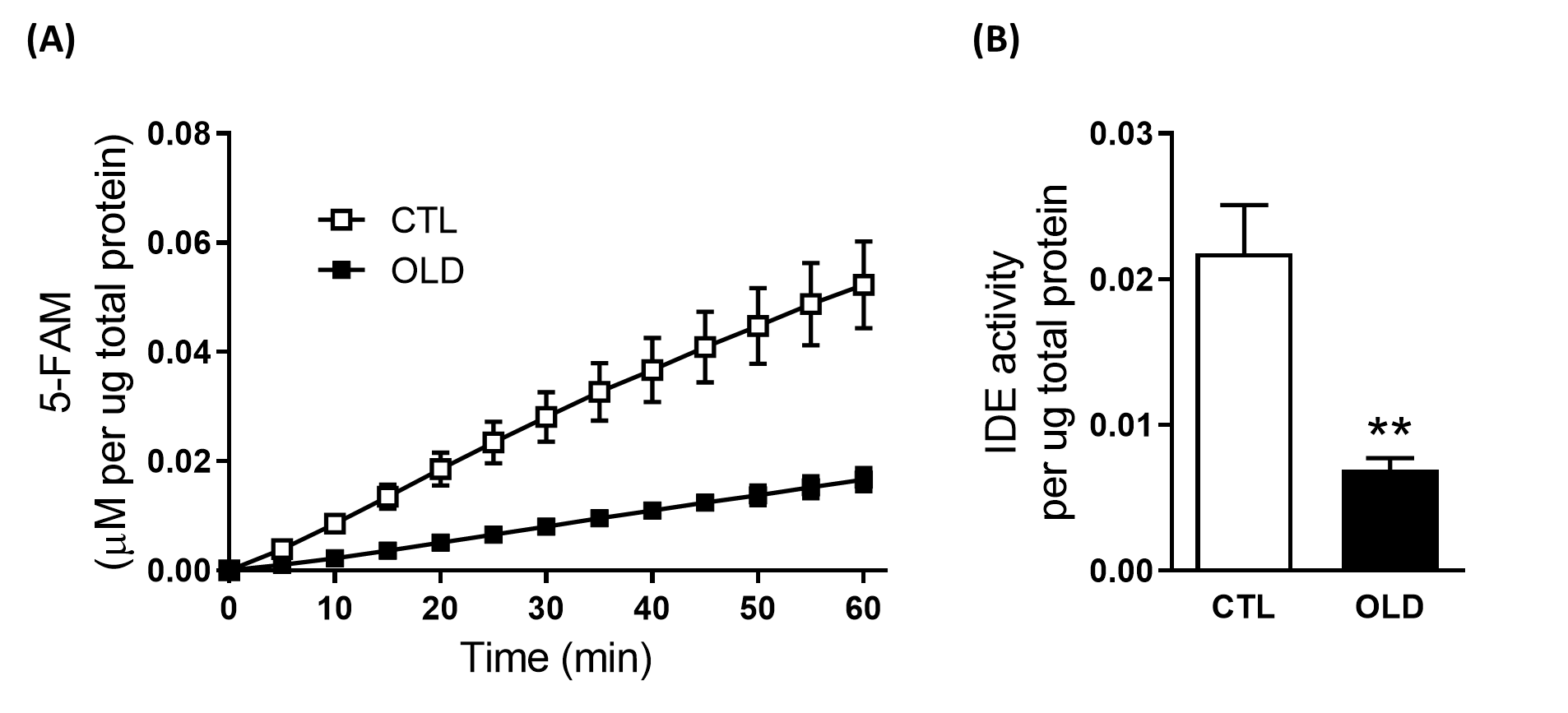


**Supplementary Figure 3.** IDE activity in the liver from 3- and 18-month-old rats. (A) Kinetics of the IDE activity assay in liver of mice. Fluorescent intensity at Ex/Em = 490/520 nm was continuously recorded, every 5 min, during 60 min. 5‐FAM concentration was calculated using a standard curve and normalized per μg of total protein. (D) IDE activity was calculated as previously described (Kurauti, Costa-Júnior, et al., 2016) and normalized per μg of total protein in the liver (n = 5 CTL and 5 OLD). CTL, 3-month-old rats; and OLD, 18-month-old rats. Data are presented as the mean ± standard deviation (SD). Student’s unpaired t-test was used to compare the groups (**p ≤ 0.01 *vs* CTL).


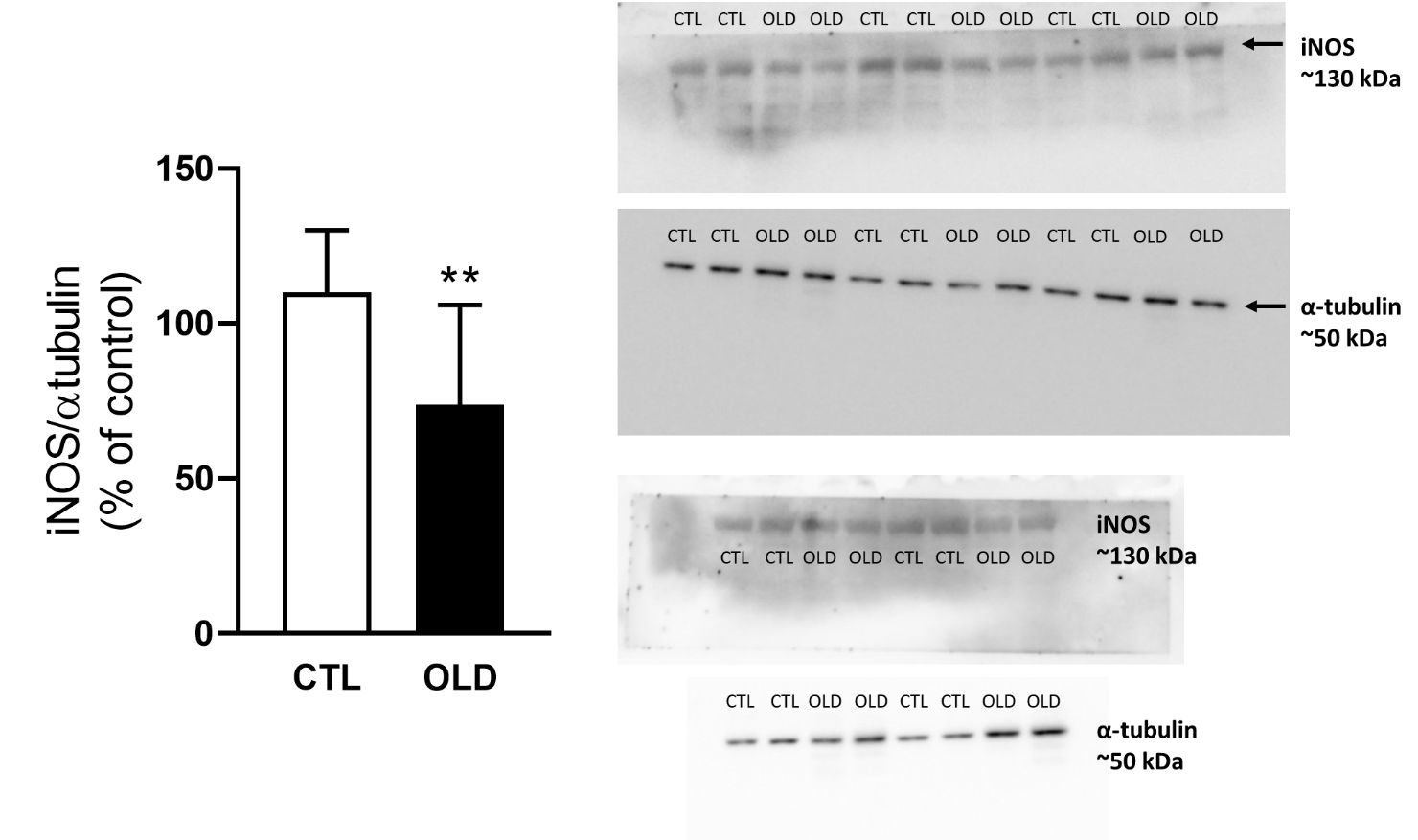


**Supplementary Figure 4.** Expression of iNOS in the liver from 3- and 18-month-old mice. Protein expression of inducible nitric oxide synthase (iNOS) in the liver and its full unedited immunoblotting images (n = 10 CTL and 10 OLD). CTL, 3-month-old mice; and OLD, 18-month-old mice. Data are presented as the mean ± standard deviation (SD). Student’s unpaired t-test was used to compare the groups (*p ≤ 0.05 *vs* CTL).
